# Black-Blood Cinematic Rendering of the Pulmonary Vasculature: A Negative-Contrast Intraluminal Preset

**DOI:** 10.3390/tomography12100143

**Published:** 2026-10-01

**Authors:** Haseeb Ur Rehman, Amy Avakian, Muhammad Umair

**Affiliations:** 1 Department of Cardiac Surgery, King Edward Medical University, Nelagumbad, Anarkali, Lahore 54000, Pakistan; 2 Department of Radiology, Southern Hills Hospital and Medical Center, Las Vegas, NV 89148, USA; 3 Department of Radiology, Columbia University, New York, NY 10032, USA; 4 Russell H. Morgan Department of Radiology and Radiological Sciences, Johns Hopkins University, Baltimore, MD 21287, USA

**Keywords:** black-blood cinematic rendering, cinematic rendering, computed tomography, pulmonary artery, pulmonary vein, opacity transfer function, three-dimensional imaging

## Abstract

Cinematic rendering (CR) is a physically based three-dimensional (3D) computed tomography (CT) visualization technique that has been applied chiefly to the external surface morphology of vascular structures. Intraluminal CR has previously been described for the cardiac chambers as black-blood cinematic rendering (BBCR), in which the opacity assigned to the contrast-enhanced blood pool is suppressed. Here, we describe the adaptation of that principle to the pulmonary vasculature as a negative-contrast intraluminal preset, in which the opacified blood pool is rendered semi-transparent so that the vessel lumen is displayed as a low-signal channel delineated by the enhancing vessel wall. We report the acquisition and rendering conditions under which the appearance was obtained in a single illustrative case performed for chest pain and showing no pulmonary vascular pathology, together with the rendering platform, base preset, and lighting configuration; the attenuation-band settings that define the appearance were tuned for the individual examination rather than applied as a fixed parameter set, were not recorded, and cannot be supplied as numerical values, so a stepwise construction procedure is given in their place. Intermediate rather than maximal compartment-specific opacification was required for the transfer function to separate lumen from wall; the case presented was acquired in an early pulmonary arterial phase, and a late split-bolus acquisition is an alternative route to the same condition that was not used here. In the case presented, the lumen of segmental and up to third-order subsegmental pulmonary arterial and venous branches was visually demonstrated on qualitative assessment by a single reader, and the preset remained stable when applied unchanged across the reconstructed 40–80% R–R window for four-dimensional display, which is a partial rather than a complete cardiac cycle reconstruction. Preset construction was less successful at the central pulmonary arteries and veins, where dense opacification and proximity to the cardiac blood pool reduced the contrast available to the transfer function. This is a single-case technical description. It demonstrates that the appearance can be produced under defined conditions and sets out the procedure by which it was constructed; it does not establish that the exact parameter values are recoverable or transferable, and it does not establish diagnostic performance, which will require prospective comparison against conventional reformats in adequately powered studies.

## Simple Summary

Computed tomography CT scans of the chest are used every day to look for clots and other problems inside the blood vessels of the lungs. Cinematic rendering is a newer way of turning that CT data into lifelike three-dimensional pictures, but until now, it has mostly been used to show the outside shape of vessels. That matters clinically because the diseases radiologists most need to find in the lung vessels—clots, tumour, scarring, and webs from old clot—sit inside the vessel, where an opaque outer surface hides them. In this report, we describe how the rendering settings can be changed so that the contrast-filled blood becomes see-through, letting the viewer look along the inside of a vessel and see its wall directly in three dimensions. We show the effect in a single patient whose lung vessels were normal, describe the scan timing and rendering settings used, and note where the approach does not yet work well. This is a technical demonstration in one case. It shows that the appearance can be produced, and it describes how, but the exact settings were adjusted by hand for this one scan and were not saved, so anyone repeating the work will have to arrive at their own settings by following the same steps. It does not show that the technique helps make diagnoses, which will require comparative studies in larger groups of patients.

## 1. Introduction

Cinematic rendering (CR) is a volumetric three-dimensional (3D) visualization technique that applies a physically based global illumination model to cross-sectional computed tomography (CT) data, producing photorealistic depictions of anatomy with naturalistic shadowing, depth cues, and surface texture [1,2,3]. In cardiovascular and thoracic imaging, CR has been applied predominantly to demonstrate the external morphology of vascular structures, cardiac chambers, implants, and devices [2,3,4,5,6,7]. Conventional contrast-enhanced renderings depict the opacified blood pool as a bright, opaque volume; the rendered surface is therefore the blood–tissue interface, and everything interior to it is hidden [2,7].

The clinical consequence of that limitation is specific to the pulmonary circulation. The abnormalities that thoracic radiologists are most often asked to identify in the pulmonary arteries are intraluminal rather than surface phenomena: acute thromboembolus; the webs, bands, intimal irregularity, and eccentric mural thrombus of chronic thromboembolic pulmonary hypertension (CTEPH); in situ thrombosis; and, less commonly, tumour embolus and intraluminal extension of pulmonary artery sarcoma [8]. On the venous side, the intraluminal question is typically tumour thrombus or the assessment of pulmonary venous anatomy before ablation or resection. Detection of these findings degrades as vessel calibre falls; segmental and subsegmental branches are precisely where visualization and interobserver agreement are weakest, even with thin-collimation multi-detector CT [9]. A rendering mode that displays the vessel interior directly, in three dimensions and with preserved branching context, is therefore of potential interest, not as a replacement for axial review, but as a means of appreciating the longitudinal extent and branch-level distribution of intraluminal disease in a single display.

The principle of suppressing the opacity assigned to the contrast-enhanced blood pool has already been established for the cardiac chambers by Rowe et al., who termed it black-blood cinematic rendering (BBCR) and demonstrated endoluminal visualization of the left ventricle, mural thrombus, and transcatheter valve implants [10]; the approach was subsequently extended to a broader range of cardiovascular applications [11]. The present report does not claim a new rendering method. It describes the adaptation of that established principle to a different anatomic target, the segmental and subsegmental pulmonary vasculature, where the governing constraint is different: unlike the cardiac chambers, the pulmonary arterial and venous compartments opacify at different times and to different degrees, so the appearance depends on contrast timing to a degree that does not apply intracardially. Our aims are to define the acquisition and rendering conditions under which this negative-contrast intraluminal appearance is obtained in the pulmonary vasculature to report the rendering platform, base preset, lighting configuration, and the stepwise procedure by which the preset was constructed, so that other investigators can follow that procedure on their own data and delineate where the approach currently fails. The report is based on a single illustrative case and is intended as a technical description, not as an evaluation of diagnostic performance.

## 2. Materials and Methods

### 2.1. Study Design and Case Selection

This is a single-case (*n* = 1) technical description. One CT examination was selected retrospectively from the clinical archive of the senior author’s institution for the purpose of illustrating the rendering technique; the examination was not acquired prospectively for this report. All still figures and the Supplementary Video derive from this same examination and the same patient. The clinical indication for the examination was chest pain, and pulmonary vascular pathology was absent. The case was selected on the grounds of diagnostic-quality opacification and absence of significant motion artefact; no systematic screening of consecutive examinations was performed, and the case is not represented as typical of unselected clinical practice. Because the examination demonstrated no pulmonary vascular pathology, the present report illustrates the rendered appearance of the normal pulmonary vasculature only; the appearance of intraluminal disease under this preset is not demonstrated.

### 2.2. CT Acquisition

The negative-contrast intraluminal appearance depends on vascular opacification being intermediate rather than maximal, so that the transfer function retains attenuation contrast between the lumen, the enhancing wall, and adjacent vasculature [1,2,10]. Two acquisition strategies produce this condition: (1) imaging in an early pulmonary arterial contrast phase, in which contrast is concentrated within the pulmonary arterial tree and has not yet equilibrated [12]; and (2) a late acquisition employing a split-bolus technique, which opacifies the pulmonary arterial and venous compartments simultaneously at differing attenuations and permits both to be assessed in a single acquisition [12,13]. The choice depends on whether arterial, venous, or combined evaluation is intended [12,13]. Split-bolus injection has separately been shown to improve visualization of the thoracic vasculature more generally [14]. The examination presented here was acquired using the first of these strategies in an early pulmonary arterial phase; a split-bolus technique was not used, and the second strategy is described here on the basis of previously published protocols rather than from experience in the present case. All figures and the Supplementary Video derive from that single acquisition. Full acquisition and contrast-administration parameters are given in Table 1.

### 2.3. Rendering Platform and Preset Construction

All renderings were generated on an independent 3D post-processing workstation using Vitrea (Canon Medical Systems Corporation, Otawara, Tochigi, Japan), software version 7.16.4.675. This platform implements physically based volumetric rendering as global illumination rendering (GIR); the term cinematic rendering is used throughout this report as the generic descriptor for this class of physically based rendering rather than as a vendor designation. Because CR output is strongly implementation-dependent, the parameter values in Table 2 should be regarded as specific to this platform and version; the underlying construction logic, suppression of opacity across the attenuation band occupied by the blood pool, as established for BBCR [10,11], is applicable to any renderer that permits opacity to be assigned as a function of attenuation, whatever the shape of the transfer-function elements that renderer provides.

**Figure 1:**
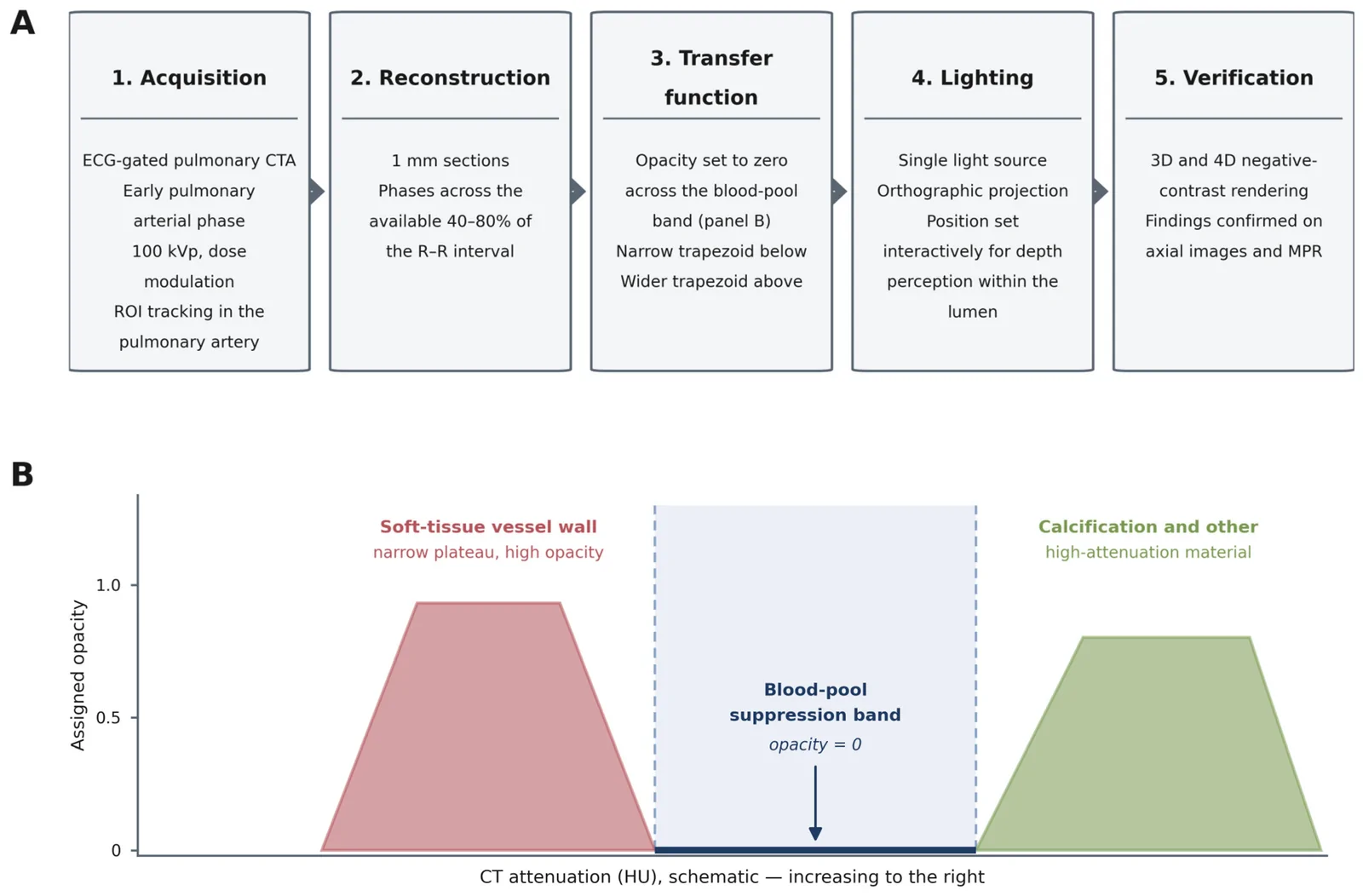
Workflow for construction and application of the negative-contrast intraluminal preset. (**A**) Sequence from acquisition through reconstruction, transfer-function modification, and lighting to the final rendering, with confirmation of every finding on the source axial images and multiplanar reformats. (**B**) Schematic of the opacity transfer function. Opacity is set to zero across the attenuation band occupied by the opacified blood pool, so that the lumen is rendered transparent; a narrow-plateau trapezoid below the band renders the soft-tissue vessel wall, and a wider trapezoid above the band preserves calcification and other high-attenuation material. The horizontal axis is schematic. The position and width of the three regions were adjusted for the individual examination for the reasons given in Section 2.3, so the panel shows the shape of the function rather than fixed attenuation values. CTA, computed tomography angiography; ROI, region of interest; MPR, multiplanar reformat.

Preset construction followed the logic established for BBCR [10,11]. Beginning from a standard contrast-enhanced CR preset, opacity was set to zero across the attenuation band occupied by the opacified blood pool, so that light is transported through the lumen rather than scattered at its surface. A narrow-plateau trapezoid was then used to assign colour and high opacity to attenuations below the blood-pool band, which is what renders the soft-tissue vessel wall; a wider trapezoid assigned colour and opacity to attenuations above the blood-pool band, preserving calcification and any high-attenuation material. Lighting was then adjusted to maintain wall conspicuity at depth within the lumen. The full sequence, and the shape of the resulting transfer function, are shown in Figure 1. The attenuation windows and trapezoid definitions that result from this process were not applied as fixed values but were adjusted for the individual examination. This is because the attenuation distribution of the opacified blood pool varies with contrast-related factors (agent, volume, injection rate, bolus chase, patient pharmacokinetics, and the point in the bolus at which the scan is triggered), with patient-related factors and with technical factors including motion, artefact, spatial resolution, and reconstruction algorithm; no single set of attenuation bands has been found to serve all examinations at this stage. Table 2 therefore reports the fixed components of the preset and the lighting values used here and identifies those components that require per-case adjustment.

A stepwise procedure is offered in place of fixed values. First, measure the attenuation of the opacified blood pool within the target vessel on the source axial images, placing the region of interest in the segmental branch of interest rather than in the main pulmonary artery, since attenuation falls with increasing branch order. For orientation only, published pulmonary CT angiographic series report main pulmonary arterial attenuation of approximately 246 ± 50 HU with left atrial attenuation of approximately 411 ± 59 HU under one split-bolus protocol [12] and approximately 410 ± 71 HU and 245 ± 25 HU for pulmonary artery and pulmonary vein, respectively, under another [13]; these figures indicate the general region in which the blood-pool band is likely to lie under a diagnostic-quality protocol, but they are not our measurements and are not offered as settings. Second, centre the zero-opacity suppression band on the measured value and widen it until the lumen renders transparent, stopping at the point at which adjacent structures of interest begin to be suppressed. Third, place the narrow-plateau trapezoid immediately below the suppression band so that the soft-tissue wall renders opaque and the wider trapezoid above it so that calcification and other high-attenuation material are preserved. Fourth, adjust lighting until the wall remains conspicuous at depth within the lumen. The endpoint of each step is visual, and the operator should expect to iterate between the second and fourth steps rather than proceeding linearly. What is reproducible from this report is therefore the procedure itself; the attenuation values arrived at in the present case are not, and a reader following these steps should expect to reach different values on their own data.

### 2.4. Definition of the Negative-Contrast Appearance

Two terms recur in this report and are worth separating at the outset. Black-blood cinematic rendering is the name of the established technique, described by Rowe et al. for the cardiac chambers [10]; negative-contrast intraluminal depiction names only the visual appearance that the technique produces. The first is the method, the second is what the image looks like, and no new technique is proposed here. The term negative-contrast intraluminal depiction is used in a strictly descriptive sense and warrants explicit definition since it could be misread. It does not denote inversion of Hounsfield values, subtraction, or any manipulation of the reconstructed CT data. No voxel values are altered. The term refers only to the rendered appearance produced when opacity is set to zero across the blood-pool attenuation band: the contrast-opacified lumen, which in a conventional rendering is the brightest and most opaque structure, is instead rendered as a transparent, low-signal channel, while the adjacent soft-tissue wall and any higher-attenuation material remain opaque. The contrast polarity of the display is therefore reversed relative to a conventional contrast-enhanced rendering, while the underlying data are unchanged. In this sense, the appearance is the pulmonary vascular equivalent of the intracardiac BBCR appearance described by Rowe et al. [10], and readers familiar with that term may regard the present preset as a pulmonary vascular application of the same principle rather than as a separate technique. We therefore treat black-blood cinematic rendering as the established designation and use it as the primary term for the technique; no new technique name is proposed here. “Negative-contrast” is retained only as a descriptor of the rendered appearance, and only because the lumen in the peripheral pulmonary vessels is not uniformly black, its residual signal varying with the degree of opacification achieved at that branch order.

### 2.5. Vessel Classification, Annotation, and Image Handling

Arterial versus venous identity was not assigned from the rendered appearance alone. Each annotated vessel was traced on the source axial images and on multiplanar reformats back to its origin, to the pulmonary arterial tree accompanying the corresponding bronchus for arteries and to the left atrium for veins, and the branch order was assigned by counting divisions distal to the segmental branch on those same source images. Segmental and subsegmental terminology follows the Fleischner Society glossary [15]. This classification was performed and verified by a single reader (M.U., a cardiovascular radiologist with 9 years of experience in radiology); no second reader was involved, and no interobserver comparison was therefore possible. All colour annotations were applied after rendering, in an external image editor, and no annotation is a product of the rendering software. Beyond the addition of these annotations, the addition of panel letters, and cropping and magnification of the inset panels, the exported images were not modified in brightness, contrast, colour, or gamma.

### 2.6. Four-Dimensional Assessment

Where time-resolved data are available, the same preset can be applied across the cardiac cycle to produce a four-dimensional (4D) CR depiction [7,16]. For the case presented, five phases at 10% increments across the 40–80% portion of the R–R interval were reconstructed, and the preset was applied unchanged to every phase; no per-phase retuning of the transfer function or lighting was performed, and the negative-contrast appearance was maintained across all reconstructed phases, as shown in Video S1. Because only the 40–80% portion of the R–R interval was available, the four-dimensional rendering presented here is a partial rather than a complete cardiac cycle reconstruction; the remainder of the cycle is not represented, and the clips should not be read as depicting the full cycle. Rendering is not instantaneous, and the time required compounds for four-dimensional work depending on the cine capabilities of the software and the computational power of the workstation. Rendering time was not measured for this examination and is not reported. Four-dimensional acquisition carries a radiation dose penalty relative to a single optimally timed phase since retrospectively gated acquisition exposes the patient across the cardiac cycle rather than during a single window. That penalty was not measured for this examination, but published cardiac CT dosimetry gives its order of magnitude. In the multicentre PROTECTION I survey of 1965 cardiac CT angiograms, prospectively triggered sequential acquisition was independently associated with a 78% lower dose–length product, electrocardiographically controlled tube current modulation with a 25% reduction, and 100 kV tube voltage with a 46% reduction [17]. These figures derive from coronary CT angiography rather than from pulmonary CT angiography, and the relative effect of each strategy may not translate exactly to pulmonary protocols, which differ in scan length, tube voltage, and gating strategy; they are cited to indicate the order of magnitude of the penalty rather than as directly transferable values. A retrospectively gated acquisition of the kind required for four-dimensional rendering should therefore be expected to carry a several-fold dose penalty over a single prospectively triggered phase, moderated by whichever dose-reduction strategies are applied; the examination presented here used tube current modulation at 100 kVp. On that basis, 4D assessment should be reserved for questions in which dynamic information is itself the object of interest, for example, phasic change in luminal calibre, or the mobility of an intraluminal filling defect, and a single optimally timed phase is sufficient for static luminal assessment.

### 2.7. Use of Artificial Intelligence

During the preparation of this manuscript, the authors used Claude (Anthropic, San Francisco, CA, USA; Opus 4.8 and Opus 5 models) to assist with language editing and restructuring of text. The tool was not used to acquire, reconstruct, render, analyse, or interpret any imaging data; to generate or modify any figure or video; or to produce any measurement, parameter value, or clinical statement reported here. All scientific content, all imaging findings, and all conclusions are the work of the authors, who have reviewed and edited the output and take full responsibility for the content of this publication.

## 3. Results

In the single case presented, the negative-contrast preset produced a transparent luminal channel bounded by an opaque wall in segmental and subsegmental pulmonary arterial and venous branches. Luminal detail was visually demonstrated up to the third-order subsegmental level. This was a qualitative assessment, made by a single reader on inspection of the renderings alongside the source images. No quantitative image-quality assessment, observer scoring, or spatial-resolution measurement was performed, and no branch-level or segment-level visibility survey was undertaken, so the consistency with which third-order detail was seen across pulmonary segments was not established. Not all pulmonary segments were assessable at this branch order. The finding is therefore an illustrative qualitative observation in one dataset and not a measured technical performance threshold; we use “visually demonstrated” rather than “resolved” for that reason. The examination showed no pulmonary vascular pathology, so the findings below describe the rendered appearance of normal pulmonary vasculature.

Luminal delineation was best in the mid and lower zone branches, where vessel calibre and course relative to the rendering plane were most favourable, and degraded toward the periphery as opacification fell with increasing branch order. No formal segment-by-segment assessability survey was undertaken, and none should be inferred from the images presented.

**Figure 2:**
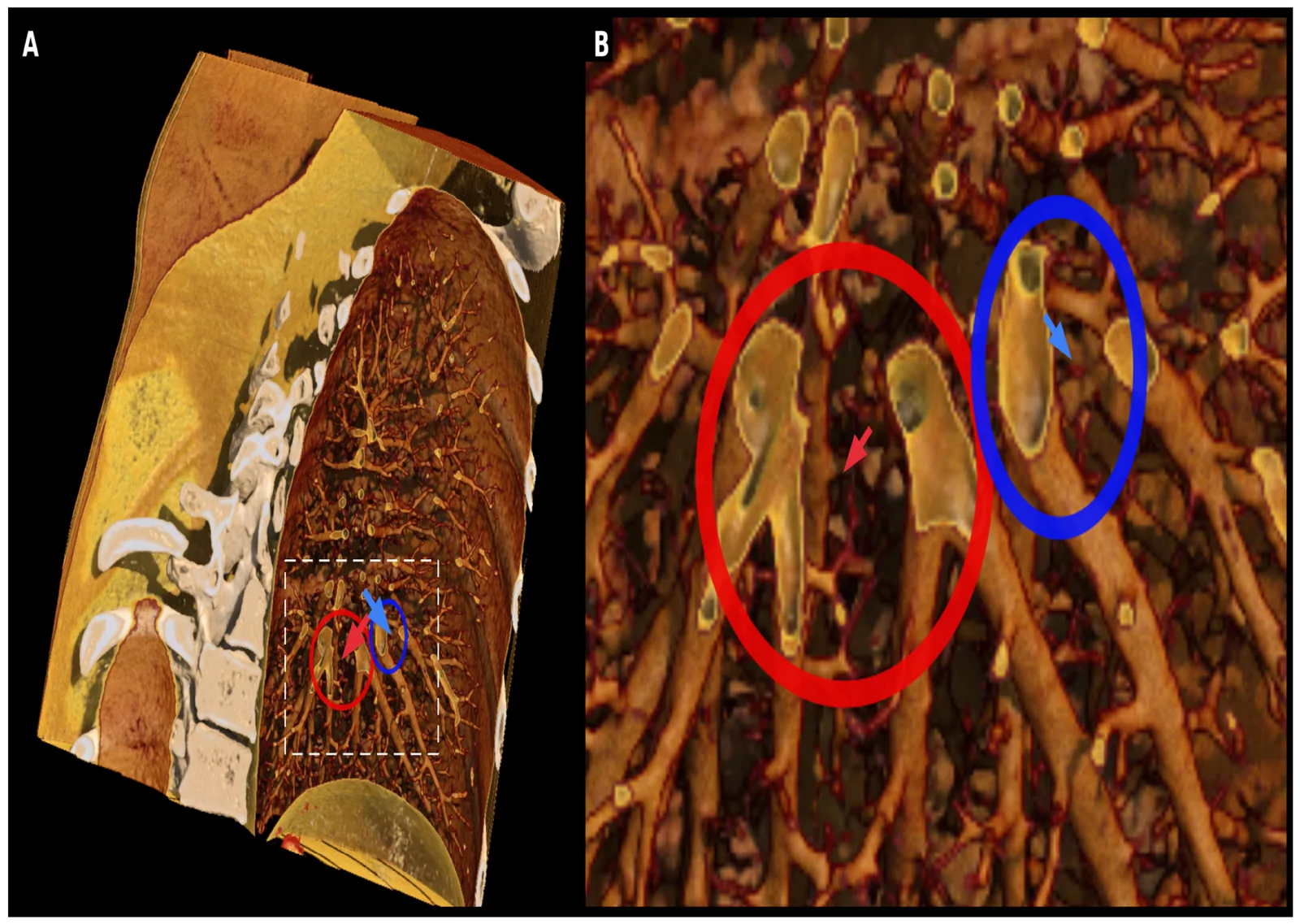
Oblique sagittal single-lung negative-contrast 3D cinematic rendering. (**A**) Overview demonstrating the segmental and subsegmental pulmonary vascular tree radiating from the hilum toward the periphery; the dashed white box indicates the region magnified in (**B**). (**B**) Magnified view of the boxed region, in which the transparent luminal channel and the opaque bounding vessel wall are directly appreciable. Blue arrow, subsegmental pulmonary artery; red arrow, adjacent subsegmental pulmonary vein. The blue and red circles in (**A**) mark the same two vessels and serve as region locators on the overview, at which scale the individual vessels are too small to be read from the arrows alone. Arterial and venous identity was assigned by tracing each vessel on the source axial images and multiplanar reformats, not from the rendered appearance. The vessels shown are left lower lobe segmental and subsegmental branches. Luminal detail is visually demonstrated up to the third-order subsegmental level in this case.

**Figure 3:**
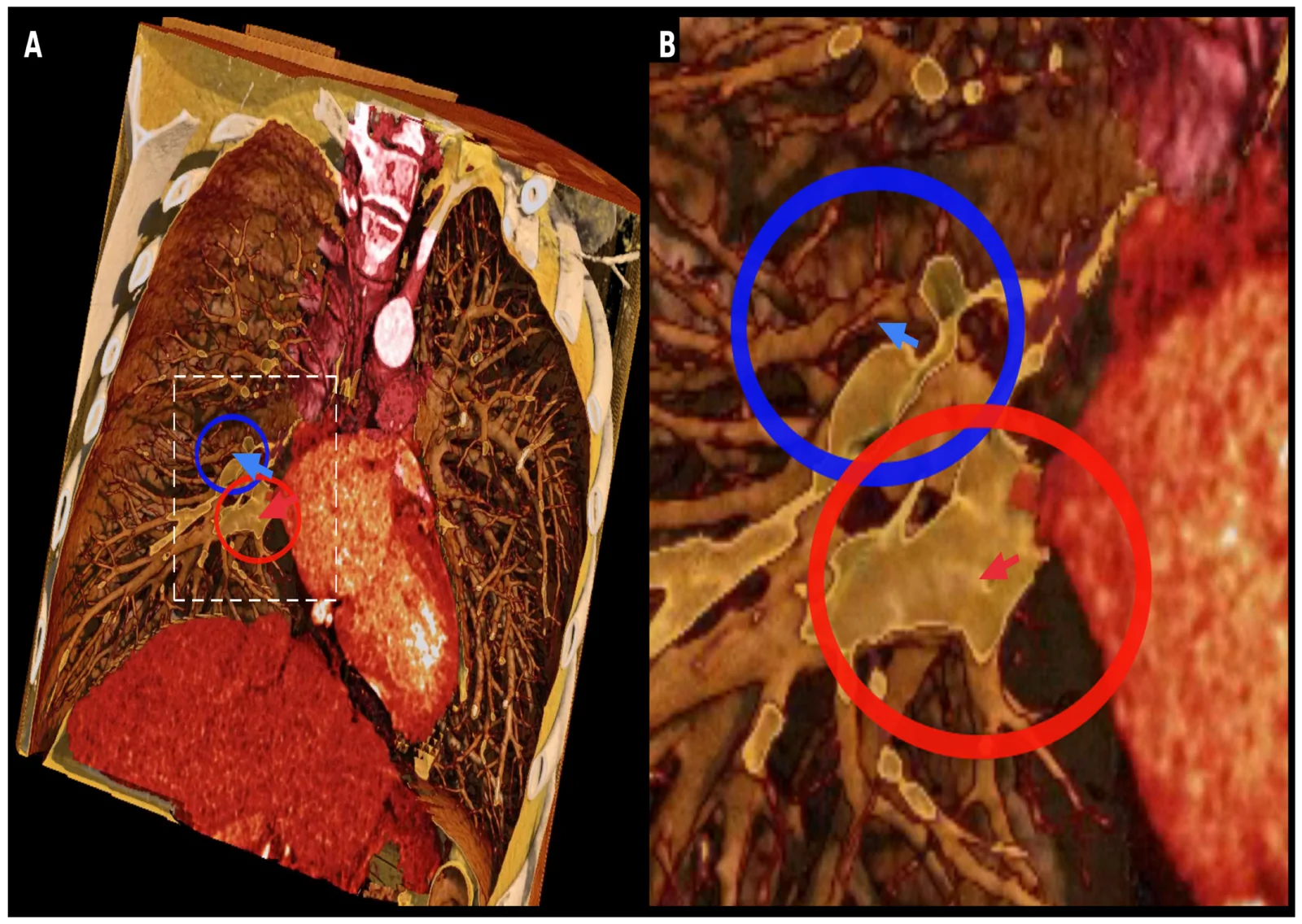
Oblique coronal negative-contrast 3D cinematic rendering of the thorax, same patient, and same acquisition as Figure 2. (**A**) Overview demonstrating the central pulmonary vasculature and bilateral segmental and subsegmental branches; the dashed white box indicates the region magnified in (**B**). (**B**) Magnified view of the boxed region. Blue arrow, segmental pulmonary artery; red arrow, corresponding segmental pulmonary vein. The blue and red circles in (**A**) mark the same two vessels and serve as region locators on the overview. Note that the negative-contrast appearance is confined to the segmental and subsegmental branches: the central pulmonary arteries retain a conventional opaque appearance, illustrating the principal technical limitation described in the Limitations section and providing, within a single image, an internal reference for the conventional opaque appearance against which the negative-contrast appearance of the peripheral branches may be compared. The magnified vessels shown are right lower lobe segmental and subsegmental branches.

An oblique sagittal single-lung projection demonstrates the branching pattern of segmental and subsegmental vessels radiating from the hilum toward the periphery, with the magnified inset showing the transparent lumen and its bounding wall at the subsegmental level (Figure 2). An oblique coronal projection demonstrates the central pulmonary vasculature together with bilateral segmental and subsegmental branches, allowing peripheral vessels to be followed against the surrounding parenchyma (Figure 3). In both projections, the negative-contrast appearance is present in the segmental and subsegmental branches but is not achieved in the central pulmonary arteries and veins, which retain a conventional opaque appearance; this is a consistent behaviour of the preset rather than an incidental feature of these projections and is considered further in the Discussion. Because both appearances are present within a single rendering, Figure 3 shows directly what the preset does and does not change: the opaque central vessels display the conventional rendered appearance of an opacified blood pool, against which the transparent segmental and subsegmental lumina can be compared. This is an internal reference rather than a matched comparison. The central and peripheral vessels differ in calibre and in degree of opacification, and no separate conventional rendering of the same vessels was generated, so the figure permits the two appearances to be seen alongside one another but does not constitute a controlled comparison of rendering methods.

Video S1. Four-dimensional negative-contrast cinematic rendering across the available cardiac phases, same patient, and same acquisition as Figure 2 and Figure 3. The clip comprises the five phases available over 40–80% of the R–R interval, looped over four consecutive playbacks; it is therefore a partial, not a complete, cardiac cycle reconstruction. The preset is applied unchanged to every phase. The clip is supplied without an audio track. The dataset shown is a different patient and a different examination from the case reported in [16].

## 4. Discussion

In the single case reported here, a CR preset built on the black-blood principle was applied to the pulmonary vasculature to produce a negative-contrast intraluminal depiction of segmental and subsegmental arterial and venous branches, and the same preset remained stable when applied unchanged across the available reconstructed phases, which spanned the 40–80% portion of the R–R interval. The principal determinant of the appearance is the interplay between contrast timing and the opacity transfer function: an early pulmonary arterial phase or a late split-bolus acquisition provides the intermediate, compartment-specific opacification that the transfer function requires in order to separate lumen from wall [2,10,12,13,14].

The relationship to the black-blood cinematic rendering of Rowe et al. deserves to be stated plainly rather than implied [10,11]. The rendering principle is the same, and we make no claim to have originated it. What differs is the anatomic target and, with it, the binding constraint. In the cardiac chambers, the blood pool is a single, uniformly and densely opacified compartment, and BBCR was described on post-contrast cardiac-gated chest CT acquired to institutional clinical standard, without a timing strategy specific to the preset [10]. The pulmonary circulation is not one compartment but two, opacifying sequentially, with attenuation falling as branch order rises; the same preset applied to an arbitrarily timed pulmonary CT angiogram will not produce the appearance because either the lumen falls outside the suppression band or the wall–lumen difference is too small for the trapezoid to exploit. The contribution here is therefore the coupling of preset design to a specific acquisition requirement, and the observation that the working range is the segmental and subsegmental tree rather than the central vessels. Framed this way, the work is a targeted extension of an established preset, not a new method, and it should be read as such.

How this display might compare with conventional post-processing is a reasonable question, and one this report cannot answer, but the expected behaviour can be stated as a testable hypothesis. Multiplanar reformation remains the reference for intraluminal assessment and has the decisive advantage of preserving quantitative attenuation, which the rendered display does not; maximum-intensity projection displays the opacified lumen well, but, by construction, obscures non-opacified intraluminal material, which is precisely what a filling defect is; minimum-intensity projection is used in the thorax chiefly for the airway and lung parenchyma, and virtual endoscopic display, although applicable to vessels, is not in routine use for the pulmonary arterial tree; and standard volume rendering shares the opaque-surface limitation of conventional CR. The plausible advantage of a negative-contrast rendering is not detection sensitivity per lesion, where multiplanar reformats (MPRs) are likely to remain superior, but the simultaneous display of longitudinal extent and branch-level distribution of intraluminal abnormality in a single image, relevant, for example, in mapping the distribution of chronic thromboembolic disease before intervention. The corresponding hypothesis, that negative-contrast CR improves the characterization of the extent of intraluminal disease without loss of per-lesion sensitivity relative to MPR, is the one that a future comparative study should be designed to test; the appropriate design is a multi-reader study with blinded, randomized presentation of MPR, maximum intensity projection (MIP), and negative-contrast CR against a reference standard, powered for the extent measure rather than for detection.

A caution about false-positive findings is warranted and should be read as a constraint on use, not a formality. Any preset that renders opacified blood semi-transparent will also render as an apparent defect anything that reduces local attenuation, whether or not it is a true filling defect: transient interruption of contrast, flow-related mixing artefact at the confluence of opacified and unopacified blood, beam-hardening, cardiac and respiratory motion, and simple failure of opacification at high branch order all produce the same rendered appearance as intraluminal material. The preset provides no attenuation value with which to discriminate between them because the display has deliberately discarded that information. Any apparent intraluminal abnormality identified on a negative-contrast rendering must therefore be confirmed on the source axial images and multiplanar reformats of the same dataset before it is reported. The technique is best understood as a display adjunct for appreciating the distribution of disease that has already been identified, and it should not be used as a primary detection tool. Applications such as CTEPH, in which the diagnostic question is the presence and extent of intraluminal disease, therefore, require this correlation as a matter of routine [8].

The chief technical limitation is at the central vessels. Constructing a reliable negative-contrast luminal preset for the central pulmonary arteries and veins proved comparatively difficult, owing to their large calibre, dense opacification, and proximity to the cardiac blood pool, all of which reduce the attenuation contrast available to the transfer function; a suppression band wide enough to render the central lumen transparent also suppresses adjacent structures of interest, so the two goals are in direct conflict at these attenuations. The technique is consequently most robust for segmental and subsegmental branches, and refinement of transfer-function design and acquisition timing for the central compartment remains necessary [2,10,12].

The conditions under which the appearance was obtained also deserve comment because they were favourable ones. The examination was selected for diagnostic-quality opacification and absence of significant motion artefact, and consecutive examinations were not screened, so the case approximates near-optimal imaging conditions and is likely to overstate how readily the preset can be constructed in unselected practice. Several factors would be expected to make construction harder. Cardiac and respiratory motion blurs the wall–lumen boundary on which the lower trapezoid depends, and the peripheral vessels of interest here are the ones most affected by it. Increased body habitus raises image noise and broadens the attenuation distribution of the blood pool, so that a suppression band wide enough to render the lumen transparent encroaches further on adjacent tissue. Reduced cardiac output delays and flattens the bolus, and suboptimal bolus timing displaces the blood pool either above the intended band or below the level at which lumen and wall can be separated at all. Heterogeneous enhancement, mixing at the confluence of opacified and unopacified blood, or a falling attenuation gradient toward the periphery, means that a single band may suit the proximal but not the distal part of the same vessel. The consequence in each case is the same: the transfer function has less attenuation contrast available to it, and the negative-contrast appearance either fails outright or becomes unreliable. How often preset construction succeeds under routine clinical conditions is unknown and cannot be estimated from a single selected case.

One hypothetical extension, raised here as speculation rather than as a near-term clinical proposal, would be a 5D CR acquisition capturing a full cardiac and a full respiratory cycle together, allowing luminal appearance to be assessed against both physiological variables at once. The radiation dose implications of acquiring both cycles are considerable, and the concept would require an independent dose-feasibility assessment before it could be entertained clinically.

It is important to distinguish clearly between what has been demonstrated, what is hypothesized, and what remains unvalidated. Demonstrated here is technical feasibility in a single case: the appearance can be produced, the construction procedure that produces it can be stated, and the appearance persists across the available reconstructed phases, spanning 40–80% of the R–R interval. Hypothesized, but not shown, is clinical utility in the depiction of intraluminal filling defects, in mapping the distribution of chronic thromboembolic disease, in the assessment of subsegmental vascular detail, and in the display of broncho-vascular relationships. Not addressed at all is diagnostic performance: no sensitivity, specificity, reproducibility, or reader-agreement data are presented, and none can be inferred from a single case. The specific questions that future work should address are whether the appearance is reproducible across scanners, vendors, and contrast protocols; whether branch-level luminal visibility can be scored reliably between observers; whether the display alters reader assessment of disease extent relative to MPR and MIP; and what false positive rate the technique carries in vessels without pathology.

### Limitations

This report has substantial limitations, which bound its interpretation closely. It rests on a single case, so no statement about generalizability across patients, body habitus, cardiac output, contrast protocols, scanner platforms, or rendering software is supportable, and variability in anatomy and pathology is entirely unrepresented. The case, moreover, showed no pulmonary vascular pathology, so the rendered appearance of intraluminal abnormality, the application for which the technique is proposed, is not demonstrated here at all. No quantitative image-quality assessment was performed: there is no branch-level visibility score, no contrast-to-noise measurement, and no spatial-resolution characterization, so the claim of third-order subsegmental visualization is a qualitative observation in one dataset. No interobserver or intraobserver evaluation was undertaken, so the reproducibility of the assessment between readers is unknown. No comparison was made against conventional volume rendering, MPR, MIP, or conventional CT angiographic review, so incremental value over existing post-processing is unestablished. The preset parameters reported are specific to one rendering platform and version, and CR output is strongly implementation-dependent, so equivalent parameters on another renderer will require empirical rederivation. The attenuation-band components were, in addition, tuned interactively for this examination rather than applied from a fixed definition, and the values arrived at were not recorded and could not later be recovered. The thin-section source dataset was subsequently no longer available to the authors, so the values could not be rederived by re-rendering, and no matched conventional cinematic or volume rendering of the same segmental and subsegmental branches at identical viewing angles could be produced for direct comparison. What is transferable from this report is therefore the construction procedure and the fixed components of the preset, not a parameter file, and a reader reproducing the appearance will have to rederive the attenuation bands for their own data, for which the stepwise procedure in Section 2.3 is intended to provide a systematic rather than an arbitrary starting point. On the basis of this experience, we would encourage investigators reporting rendering presets to save the transfer-function parameter file or, at minimum, a screenshot of the transfer-function settings, as part of the study record; doing so as a matter of routine would make technical notes of this kind substantially more reproducible across sites. The four-dimensional assessment rests on a partial (40–80% R–R) reconstruction, so stability of the appearance across the remainder of the cardiac cycle is untested. Finally, and most importantly, diagnostic performance cannot be established from a single illustrative case, and the potential applications discussed above should be read as hypotheses for evaluation rather than as demonstrated capabilities.

## 5. Conclusions

In a single illustrative case, a cinematic rendering preset built on the black-blood principle and combined with an early pulmonary arterial phase acquisition produced a negative-contrast intraluminal depiction of the pulmonary vasculature that was visually demonstrated, on qualitative assessment by a single reader, up to the third-order subsegmental level, and remained stable across the reconstructed 40–80% portion of the cardiac cycle. Preset design for the central pulmonary arteries and veins remains an unsolved technical problem. This is a feasibility demonstration rather than an established technique, and the appearance should be interpreted alongside the source axial images in every case. In practical terms, the preset is best applied after standard CT angiographic review, in selected cases, to illustrate the extent and branch-level distribution of disease that has already been identified, rather than as a primary means of detection. The immediate next step is a prospective multi-case series applying the preset, with the platform, base preset, lighting configuration, and construction procedure reported here, to patients with and without known pulmonary vascular disease, with blinded branch-level scoring against multiplanar reformation, a study that is directly actionable with the procedure described here.

## Figures and Tables

**Table 1:** CT acquisition and contrast-administration parameters for the case presented. All figures and Video S1 derive from this single examination.

Parameter	Value
Number of examinations/patients	1/1
Acquisition strategy used	Early pulmonary arterial phase
Scanner manufacturer and model	Revolution Apex Select (GE HealthCare, Chicago, IL, USA)
Detector configuration	64 × 0.625 mm, 40 mm *z*-axis coverage
Tube voltage (kVp)	100
Tube current/dose modulation	Tube current modulation enabled
ECG gating	Yes; five phases reconstructed at 10% increments over the 40–80% R–R window
Reconstructed slice thickness/increment (mm)	1 mm
Reconstruction kernel	Sharp vascular kernel
Contrast agent and iodine concentration	Iohexol 350 mg I/mL (Omnipaque 350, GE HealthCare, Chicago, IL, USA)
Total contrast volume (mL)/injection rate (mL/s)	60 mL at 4–6 mL/s
Split-bolus ratio and inter-bolus delay	Not applicable; split bolus not used
Scan trigger method and delay	Automated region-of-interest bolus tracking, region of interest in the pulmonary artery, threshold 130 HU; acquisition initiated automatically on threshold, with no operator-set delay

Image data were transferred from the scanner listed above to an independent 3D workstation on which all post-processing was performed (Section 2.3). ECG, electrocardiographic; HU, Hounsfield units.

**Table 2:** Negative-contrast intraluminal cinematic rendering preset parameters as applied in the case presented.

Preset Component	Setting in the Case Presented	Transferability
Base preset from which the preset was derived	Lung Vessel GIR	Fixed component of the preset
Zero-opacity suppression band across the blood pool	Centred on the attenuation of the opacified blood pool; position and width set interactively (Section 2.3)	Requires case-specific adjustment; values not recorded
Narrow-plateau trapezoid rendering the soft-tissue vessel wall	Placed immediately below the suppression band; set interactively	Requires case-specific adjustment; values not recorded
Wider trapezoid preserving calcification and high-attenuation material	Placed above the suppression band; set interactively	Requires case-specific adjustment; values not recorded
Lighting preset and number of light sources	GIR Upper Right Light; single light source	Fixed component of the preset
Ambient, diffuse, and specular values	Ambient 0.43, diffuse 0.30, specular 0.26	Reported for the case presented; adjustable
Key light position	Set interactively for each rendering by moving the source through the scene while inspecting the resulting shadowing and depth cues over the structure of interest	Requires case-specific adjustment
Light projection	Orthographic	Fixed component of the preset

GIR, global illumination rendering. Values are specific to the rendering platform and version stated in Section 2.3, and the lighting values are those used in the case presented. Components marked as requiring case-specific adjustment were tuned interactively for this examination, were not recorded, and cannot be supplied as numerical values; the procedure by which they were derived is set out in Section 2.3 and its consequences in the Limitations section.

## Data Availability

The imaging data underlying this technical note are not publicly available in order to protect patient privacy. The thin-section source dataset from which the renderings were generated is no longer accessible to the authors and cannot be shared or reprocessed.

## Supplementary Materials

The following supporting information can be downloaded at: https://www.mdpi.com/article/10.3390/tomography12100143/s1, Video S1: Four-dimensional cinematic rendering clip applying the negative-contrast preset across the available 40–80% of the R–R interval, supplied without an audio track.
